# Structural Changes in Stx1 Engineering Monoclonal Antibody Improves Its Functionality as Diagnostic Tool for a Rapid Latex Agglutination Test

**DOI:** 10.3390/antib7010009

**Published:** 2018-02-01

**Authors:** Daniela Luz, Emerson A. Shiga, Gang Chen, Wagner Quintilio, Fernanda B. Andrade, Andrea Q. Maranhão, Bruna A. Caetano, Thaís Mitsunari, Míriam A. Silva, Letícia B. Rocha, Ana M. Moro, Sachdev S. Sidhu, Roxane M. F. Piazza

**Affiliations:** 1 Laboratório de Bacteriologia, Instituto Butantan, São Paulo, SP 05503-900, Brazil; daniedaluz@yahoo.com.br (D.L.); emerson.shiga@butantan.gov.br (E.A.S.); fernanda.andrade@butantan.gov.br (F.B.A.); bruna.caetano@butantan.gov.br (B.A.C.); thais.mitsunari@butantan.gov.br (T.M.); miriam.silva@butantan.gov.br (M.A.S.); leticia.rocha@butantan.gov.br (L.B.R.); 2 Department of Molecular Genetics, Terrence Donnelly Centre for Cellular and Biomolecular Research, University of Toronto, Toronto, ON M5S 3E1, Canada; gchen2012@gmail.com (G.C.); sachdev.sidhu@utoronto.ca (S.S.S.); 3 Laboratório de Biofármacos em Células Animais, Instituto Butantan, São Paulo, SP 05503-900, Brazil; wagner.quintilio@butantan.gov.br (W.Q.); ana.moro@butantan.gov.br (A.M.M.); 4 Laboratório de Imunologia, Universidade de Brasília, Brasília 70910-900, Brazil; andreaqm@unb.br

**Keywords:** antibody, scFv, Stx1, STEC

## Abstract

Stx1 toxin is one of the AB_5_ toxins of Shiga toxin-producing *Escherichia coli* (STEC) responsible for foodborne intoxication during outbreaks. The single-chain variable fragment (scFv) is the most common recombinant antibody format; it consists of both variable chains connected by a peptide linker with conserved specificity and affinity for antigen. The drawbacks of scFv production in bacteria are the heterologous expression, conformation and stability of the molecule, which could change the affinity for the antigen. In this work, we obtained a stable and functional scFv-Stx1 in bacteria, starting from IgG produced by hybridoma cells. After structural modifications, i.e., change in protein orientation, vector and linker, its solubility for expression in bacteria was increased as well as the affinity for its antigen, demonstrated by a scFv dissociation constant (*K*_D_) of 2.26 × 10^−7^ M. Also, it was able to recognize purified Stx1 and cross-reacted with Stx2 toxin by ELISA (Enzyme-Linked Immunosorbent Assay), and detected 88% of Stx1-producing strains using a rapid latex agglutination test. Thus, the scFv fragment obtained in the present work is a bacteria-produced tool for use in a rapid diagnosis test, providing an alternative for STEC diagnosis.

## 1. Introduction

The single-chain variable fragment (scFv) is a common format of recombinant antibody fragments, and consists of heavy (VH) and light (VL) variable domains, retaining the specificity of parental immunoglobulin [1]. Successful construction of scFvs depends on the choice of the peptide linker and connection of the VH and VL domains, which affect the stability and recognition properties of these antibody molecules [2,3]. Improvements in engineered recombinant antibody fragments could lead to ideal tools for therapy and diagnosis [1]. Indeed, it is possible to increase the antigen-binding affinity and specificity by mimicking somatic hypermutation during an immune response [4]. Moreover, with recombinant antibody technology, it is possible to replace animal immunization and hybridoma development with a bacterial system, which is capable of synthesizing and expressing practically unlimited quantities of antibodies, which would provide for a more cost-effective diagnostic tool [5]. 

Shiga toxin 1 (Stx1) is a potent bacterial toxin produced by Shiga toxin-producing *Escherichia coli* (STEC). It is a member of the AB_5_ bacterial toxin family, of which the B subunit (StxB) binds to globotriaosylceramide receptors (Gb3) on the host cell membrane and translocate the active A subunit (StxA) into the cytosol. StxA exhibits RNA *N*-glycosidase activity towards 28S rRNA, resulting in inhibition of protein synthesis in eukaryotic cells. This inhibition is associated with the ability of STEC bacteria to cause hemolytic uremic syndrome (HUS) in humans [6,7,8,9]. Even though the impact of foodborne illness on a global scale is difficult to estimate, the spread of disease through contaminated food still plays a major role in mortality, raising the importance of the standardization of rapid diagnostic methods, to minimize economic costs in terms of productivity loss, incomes, and health care [10]. 

Herein, we report the construction of a monoclonal scFv fragment targeting Stx1 by antibody engineering for diagnostic application. We demonstrated here the importance of testing different gene assembly and cloning strategies to obtain a functional scFv fragment, since a recombinant antibody can sometimes lose its affinity for the antigen after purification. The resulting molecule was able to bind purified antigen, and recognize toxin-producing strains using a rapid latex agglutination test, and thus it is considered a promising tool for STEC diagnosis.

## 2. Materials and Methods

### 2.1. scFvStx1(I) Gene Design, Expression and Purification

**Figure 1:**
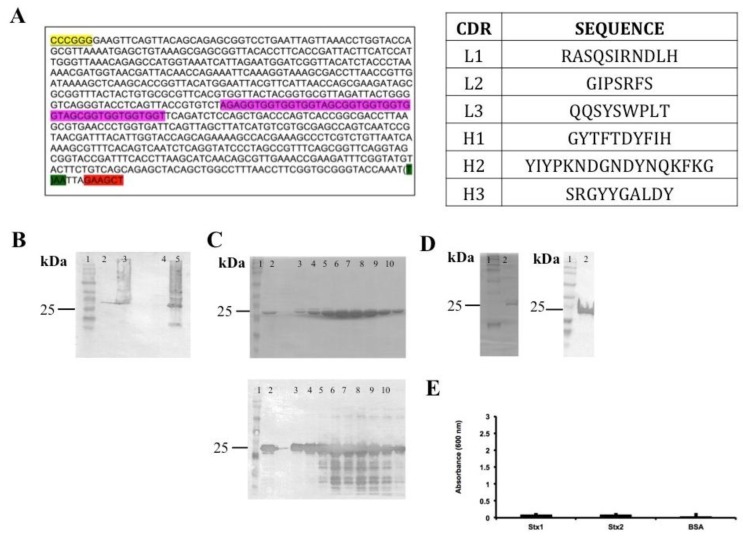
Summary of scFvStx1(I) cloning and procurement. (**A**) Gene sequence and identification of variable chain CDR (complementarity-determining regions). Restriction enzyme *BamHI* site is highlighted in yellow, *HindIII* site is highlighted in red, linker sequence is highlighted in pink and a stop codon is highlighted in green; (**B**) Immunoblotting membrane with anti-6XHis-tag analysis of scFvStx1(I) recombinant protein expression induction: molecular marker (lane 1); fraction before induction (lane 2); fraction post-induction (lane 3); soluble fraction (lane 4); insoluble fraction (lane 5); (**C**) 12% SDS-PAGE analysis (above) of scFvStx1(I) recombinant protein affinity purification and mirror immunoblotting membrane with anti-6XHis-tag (below): molecular marker (lane 1); sample flow through (lane 2); imidazole-eluted fractions (lanes 3 to 10); (**D**) 12% SDS-PAGE analysis (**left**) of scFvStx1(I) recombinant protein after dialysis, and mirror immunoblotting membrane with anti-6XHis-tag (**right**): molecular marker (lane 1); post-dialysis sample (lane 2); (**E**) ELISA for cross-reaction employing 2 μg of each toxin and 0.2% BSA (bovine serum albumin) as control. scFvStx1 was used at 30 μg/mL concentration and the detection antibody was peroxidase-conjugated anti-His-tag (SIGMA). The assay was performed in triplicate.

The scFvStx1(I) gene was constructed on the basis of murine hybridoma (mAb 3E2) secreting Stx1 IgG monoclonal antibodies [11], using the same protocol as described by Luz et al. [12]. The DNA encoding the scFvStx1(I) fragment was designed using the BioEdit program (www.bioedit.com) in a VH-linker-VL orientation, using a regular (Gly4Ser)3 linker type, and synthesized by GenScript (Jiangsu, China), (Figure 1). This gene was first cloned into the pAE vector through restriction enzymes BamHI/HindIII (Thermo Scientific, Waltham, MA, USA). Cloning was performed using T4 ligase (Invitrogen, Carlsbad, CA, USA), following the manufacturer’s recommendations and transformed into E. coli BL21 (DE3) (Promega, Madison, WI, USA) competent cells [13]. The recombinant vector was confirmed by plasmid sequencing. The scFvStx1(I) expression was induced by the addition of IPTG to 1 mM (Invitrogen). The purification was performed under denaturing conditions by the addition of 8 M urea by IMAC chromatography on AKTA Primeplus (GE Healthcare, Uppsala, Sweden), using a His-Trap HP Ni Sepharose column (GE Healthcare, Uppsala, Sweden). The first purified scFvStx1(I) was dialyzed to reestablish protein conformation with a Slide-A-Lyser Dialysis G2 (Thermo Scientific, USA) against decreasing concentrations of urea (5, 3, 2, 1 and 0.5 M). The purified protein was analyzed by SDS-PAGE and immunoblotting, detected by HRP-conjugated anti-His-tag monoclonal antibody (1:5000) (Sigma, St. Louis, MO, USA). 

### 2.2. scFvStx1(I) Gene Modifications 

**Figure 2:**
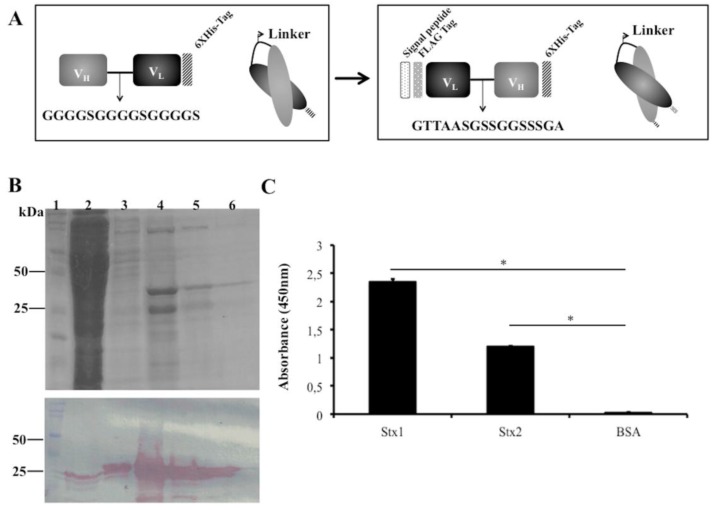
scFvStx1 molecule designs and purified protein. (**A**) Schematic representation of scFvStx1 assembly and modifications; (**B**) 12% SDS-PAGE analysis of scFvStx1 recombinant fragment affinity purification. Molecular marker (lane 1); flow-through fraction (lane 2); wash fraction (lane 3); elution fractions with 20 to 40 mM imidazole (lanes 4–6) showing a 25-kDa protein. Below, immunoblotting detected by peroxidase-conjugated anti-Flag antibody (1:5000), showing detection of a 25-kDa protein; (**C**) ELISA for cross-reaction employing 2 μg of each toxin and 0.2% BSA as control. scFvStx1 was used at EC50 concentration and peroxidase-conjugated anti-Flag antibody (SIGMA) was used for detection. The assay was performed in triplicate and considered positive when *p* > 0.05 by Student’s *t*-test versus control (*).

For the second scFv arrangement gene, the DNA fragments encoding the corresponding VL and VH domains were amplified from the previous synthetic gene, using as primers: VLFw (5′ CCT ATG CAT CCG ATT ACA AAG ATG ACG ATG ACA AAG GCG GTG ATA TCC AGC TGA CCC AGA G 3′), VLRv (5′ CTG CCA CCA CTA CTA CCA CTA GCG GCA GTA GTA CCC TTC AGT TCT AAT TTG GTA CC 3′), VHFw (5′ GTG GTA GTA GTG GTG GCA GTA GCA GTG GTG CCG AAG TTC AGT TAC AGC AGA GC 3′) and VHRv (5′ TTG TCG GCC GAA GAC ACG GTA ACT GAG GTA C 3′). The resulting gene was designated *scFvStx1,* and for this construction, the orientation was VL-Linker-VH, while the linker was also changed (Figure 2)*.* Both *scFvStx1* DNA and the pscFvHis-MBP [14] vector were double-digested with NsiI and EagI (NEB, Knowl Piece, Wilbury Way, Hitchin, UK) and purified with Qiaquick PCR purification (Qiagen, Hilden, Germany). Cloning was performed using T4 ligase (Invitrogen), following the manufacturer’s recommendations and transformed into *E. coli* BL21 (DE3) pLysS (Promega, Madison, WI, USA) competent cells [13]. The recombinant vector was confirmed by plasmid sequencing, and the final construction was designated p*scfvStx1*.

### 2.3. scFvStx1 Fragment Procurement 

The scFvStx1 fragment was obtained from p*scFvStx1* construction. Gene expression and purification was performed as described by Luz et al. [12]. Briefly, gene expression was induced by the addition of IPTG to 0.01 mM, and purification performed by IMAC chromatography. The purified protein was analyzed by SDS-PAGE and immunoblotting, with detection by HRP-conjugated anti-His-tag monoclonal antibody (1:5000) (Sigma, St. Louis, MO, USA). Affinity was determined by surface plasmon resonance (BIAcore T200, GE Healthcare, Uppsala, Sweden) following the manufacturer’s recommendations. Briefly, Stx1 (purchased from Tufts University School of Medicine, Boston, MA, USA) at 5 µg/mL was immobilized in 10 mM sodium acetate buffer, pH 5.5 (152 RU) on CM5 sensor chips activated by mixing equal amounts of *N*-ethyl-*N*′-(dimethylaminopropyl) carbodiimide (EDC) and *N*-hydroxysuccinimide (NHS). Analyses were done using the BIAcore T200 (GE Healthcare, Uppsala, Sweden) instrument. The running buffer used was HBS-EP buffer, pH 7.4, containing 10 mM HEPES, 150 mM NaCl, 3 mM EDTA, and 0.05% Tween 20. All samples were prepared in HBS-EP buffer (0–7.4 µM, twofold dilutions), and the kinetic study was carried out in a multicycle model at 25 °C and a flow rate of 30 µL/min (contact of 120 s and dissociation of 600 s). Between cycles the sensor chip was regenerated by a 15 µL pulse of 100 mM glycine containing 2 mM MgCl_2_, pH 2. The kinetic affinity constant (*K*_D_) was calculated using BIAevaluation version 3.0 (GE Healthcare, Uppsala, Sweden), using the Langmuir 1:1 binding model. The experiments were performed in duplicate. For the effective binding concentration EC50, the antibody concentration required to reach half of the maximal absorbance in ELISA [12] was determined. This concentration was used to perform the cross-reactive ELISA using both Stx toxins (2 μg/mL) immobilized in a 96-well plate using 0.2% BSA as control. For ELISA assays, 0.2% BSA was used as blocking solution for 1 h at room temperature, and the same conditions were used in the following incubations, such as the scFvStx1 and the HRP-conjugated anti-His-tag monoclonal antibody (1:5000, diluted in blocking solution). Between each step, the plates were washed three times with PBS-0.05% Tween 20. Student’s *t*-test was used for statistical analysis, where the differences were considered statistically significant when *p* ≤ 0.05.

### 2.4. Rapid Latex Agglutination Test (RALT)

RALT was performed as described by Ristori et al. [15], with modifications. Briefly, the latex suspension was incubated with scFvStx1 (500 μg/mL) for 18 h at room temperature, followed by two blocking steps (with 0.2 M ethanolamine and with 1% BSA) for conjugation. After the blocking steps, the sample was maintained in stock buffer. As samples for agglutination tests, logarithmic phase (OD 0.5–0.8) lysates of 23 STEC strains were used as positive controls [11] and lysates of enteropathogenic *E. coli*, enterotoxigenic *E. coli*, and *Shigella flexneri* as negative controls. The bacterial cells were cultured in *E. coli* broth enriched with 0.5 ng/mL ciprofloxacin for 4 h at 37 °C, and then lysed with Triton X-100 for 1 h at 37 °C. The assay was performed on a glass slide, using a mixture of 20 μL of scFvStx1-latex and 20 μL of bacterial lysate, checking for agglutination after 1 min of gentle mixing. Three independent experiments were performed. 

## 3. Results

The DNA encoding the variable domains of both heavy and light chains were isolated from mRNA extracted from hybridoma cells (mAb 3E2) [11] and used for scFv fragment design (Figure 1A). The first scFv design/construct was the VH-linker-VL, using (Gly4Ser)3 as the linker for the *scFvStx1(I)* gene. Even after several attempts, this construction resulted in inclusion body expression and non-functional protein afterwards (Figure 1). 

As an alternative to increasing scFv stability during production, we changed the scFvStx1(I) design by adopting another linker (GTTAASGSSGGSSSGA), a linker identified from a phage library designed to optimize the linker between VL and VH of an anti-MBP scFv. In addition, the orientation of the variable domains was reversed as VL-linker-VH. A Ptac-driven expression vector that harbors a signal peptide sequence for periplasmic expression was used for scFv expression, the new gene was designated *scFvStx1* (Figure 2A). 

scFvStx1 was purified using a nickel affinity column (IMAC), yielding 2 mg/L, resulting in a 25-kDa protein on SDS-PAGE and immunoblotting (Figure 2B). The affinity constant (*K*_D_) was determined by SPR as 2.26 × 10^−7^ M. The half-maximal effective concentration (EC50) was determined to be 600 nM by ELISA, and this concentration was used to test cross-reactivity with the homologous toxin Stx2. scFvStx1 was able to bind to both toxins with significant difference compared to the control (Figure 2C). Compared with the parental IgG monoclonal antibody, scFvStx1 was more rapidly obtained in a bacterial system, remaining with the same yield and cross-reactivity (Table 1). 

**Figure 3:**
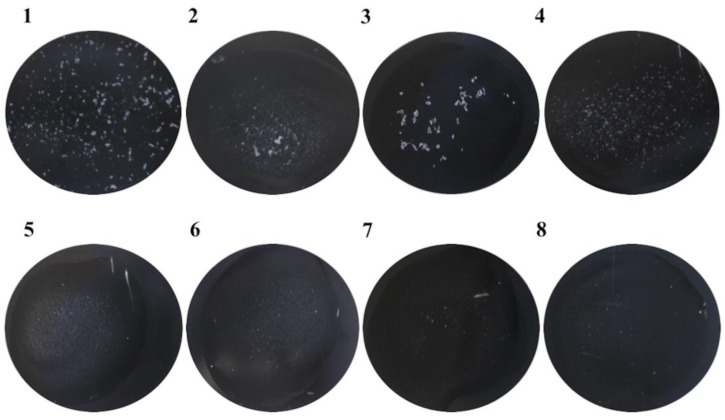
Representative example of the rapid latex agglutination test, with scFvStx1, against positive and negative isolates. The figure represents a 1-min reaction. (**1**) STEC O111:NM (human origin); (**2**) STEC O26:H11 (animal origin); (**3**) STEC O111:H8 (human origin); (**4**) STEC O157:H7 (human origin); (**5**) enterotoxigenic *Escherichia coli* (ETEC); (**6**) enteropathogenic *Escherichia coli* (EPEC); (**7**) *Shigella flexneri*; (**8**) *E. coli* broth.

Moreover, scFvStx1 was conjugated with latex particles and employed in a rapid latex agglutination test. After a 1-min reaction, the recombinant fragment recognized 88% of the 23 Stx1-producing isolates (STEC) tested. Figure 3 shows a representative agglutination assay. In addition, the scFvStx1-latex complex was stable even after 2 months, giving the same percentage of detection.

## 4. Discussion

STECs are foodborne pathogens responsible for 90% of HUS cases [16]. The Shiga toxins produced by these strains are the main targets for detection [17]. Although commercial tests are available for Stx detection, none of them use a recombinant antibody produced in bacteria [18], which could decrease the cost of the final product, since this product is costly which makes it difficult to use in every routine laboratory. Here, we developed a recombinant scFv antibody fragment against Stx1 produced in bacteria, which has a potential for a rapid STEC diagnosis. 

The most important challenges in protein engineering are to determine which factors influence recombinant protein stability and functionality [19]. The first strategy used to obtain the scFvStx1(I) fragment resulted in a protein occurring as inclusion bodies, and consequently, the purification was performed under denaturing conditions. The refolding process resulted in many aggregates, which interfered with functionality; this could have been due to the fact that under cytoplasmic reducing conditions, the intra-domain disulfide bonds of conserved antibody domains cannot form, which can interfere with the stability of purified scFv [20,21]. As reviewed by Worn [19], the highly conserved intra-domain disulfide bonds are critical for the stability of scFv fragments [22,23], where only intrinsically very stable scFv fragments will be able to fold correctly in sufficient amounts to be active as cytoplasmic intrabodies. This is also consistent with the finding that many cytoplasmic intrabodies show low expression levels and short half-lives [24]. 

Thus, to overcome the non-functional insoluble molecule, we made some changes in the *scFvStx1(I)* gene (then designated *scFvStx1*), in which the orientation was changed to VL-linker-VH. This arrangement allowed the CDH3 to be free at the C-terminal end, since this CDR is likely the most important contributor to antigen binding for most natural antibodies [25]. In addition, based on the fact that the linker could also interfere with molecule stability during expression or storage [26,27], the linker that connects the VL and VH domains was optimized for a common framework by phage display technology (data not shown). Compared with the classic Gly4Ser linker, this linker, GTTAASGSSGGSSSGA, was less flexible and more hydrophilic. Less flexible linkers with proline, threonine and alanine increase stability and the biological activity of recombinant proteins [27]. 

The *scFvStx1* gene was also cloned into a more specific expression vector for scFv expression [14], which contained besides the histidine tag (6XHis) for nickel affinity chromatography, a FLAG^®^ tag (Thermo Scientific, Waltham, MA, USA), which is composed of 8 hydrophilic amino acids (N-Y-K-N-N-N-K). FLAG^®^ tag (Thermo Scientific, Waltham, MA, USA) is a small tag that does not interfere with protein conformation and activity, but it does increase detection sensitivity 20- to 200-fold compared with His-tag. The expression of this recombinant antibody was driven to host cell periplasm. Unlike the cytoplasm, the periplasm is an oxidative environment, and thus, it is more suitable for disulfide bond formation, which improves the stability of the molecule [28]. 

The new scFvStx1 was expressed with a yield of 2 mg/L, in a soluble manner, with a *K*_D_ of 2.26 × 10^−7^ M, lower than its precursor mAb 3E2 [11]. Differences in the affinity constant of mono or dimeric antibodies are due to avidity [29]. The recombinant antibody affinity constant is usually lower compared to IgG molecules, which was previously demonstrated by our group with the scFv anti-Stx2 [12]. Moreover, the scFv purification process resulted in a less expensive and faster protocol compared to that for parental IgG (Table 1). scFv-Stx1 also showed cross-reactivity with both toxins, as observed with the scFvStx2 [12] constructed on the basis of Stx2 mAb, which showed cross-reactivity as well [11]. Additionally, scFv was able to detect Stx1-producing isolates with the rapid latex agglutination test. This test is a possible alternative for the current commercial tests. 

Taken together, these results strengthen the importance of using structural and expression strategies to improve the functionality and stability of scFv molecules, which contributes to the development of scFv fragments for use in identifying the agent of a worldwide foodborne outbreak, such as STEC. 

## Figures and Tables

**Table 1:** Comparison of scFvstx1 and IgG parental antibody. IgG characteristics were obtained from Rocha et al. (2012).

	scFvStx1(II)	IgG mAb
**Affinity constant (*K*_D_)**	2.26 × 10^−7^ M	2.5 × 10^−10^ M
**Expression system**	Bacterial	Hybridoma
**Yield after purification**	2 mg/L	2 mg/L
**Time to obtain (weeks)**	1	5–7
Cross-reactivity to Stx2 *	Yes	Yes

* Tested by ELISA.
